# Inflammasomes meet organoids and artificial intelligence: unraveling the complexity of gynecological inflammation

**DOI:** 10.3389/fimmu.2026.1753651

**Published:** 2026-04-17

**Authors:** Lanyue Zhang, Jiangnan Zhao, Guanghui Cheng, Jiaxin Wu, Yunhao Liu, Guo Lei, Qin Wang

**Affiliations:** 1 Clinical Medical College, Southwest Medical University, Luzhou, China; 2 Sichuan Provincial Center for Gynecology and Breast Diseases (Gynecology), Affiliated Hospital of Southwest Medical University, Luzhou, China

**Keywords:** artificial intelligence (AI), chronic inflammation, gynecological diseases, inflammasome, innate immunity, multi-omics integration, organoid, TME

## Abstract

Gynecological diseases represent a persistent global health burden. According to a WHO report, the global incidence of gynecological diseases exceeds 65%. Furthermore, over 90% of women suffer from gynecological issues to varying degrees. They account for about 4.5% of the global disease burden. This share is higher than that of other major global health problems. For example, malaria accounts for about 1.04%. Tuberculosis accounts for about 1.9%. Ischemic heart disease accounts for about 2.2%. Mounting evidence suggests that inflammasomes act as master regulators linking chronic inflammation with the onset and progression of gynecological disorders including endometriosis, ovarian cancer, and polycystic ovary syndrome. Yet, traditional two-dimensional cultures and animal models fail to reproduce the intricate immune–endocrine microenvironment of the human reproductive system, impeding translational progress. This review provides an integrated perspective that unites inflammasome biology, organoid technology, and artificial intelligence (AI) into a new research paradigm for precision immunology. We comprehensively summarize the molecular mechanisms of inflammasome activation—particularly NLRP3, AIM2, and IFI16—and their dual roles in inflammatory injury and immune regulation across gynecological diseases. We further highlight advances in organoid-based models that reconstruct the three-dimensional architecture and immune context of reproductive tissues, offering a physiologically relevant platform for mechanistic exploration. In parallel, AI-driven multi-omics analytics and image-based deep learning are shown to accelerate data interpretation, reveal inflammasome-related regulatory networks, and optimize experimental design. By bridging immunopathology, bioengineering, and computational intelligence, this review establishes a conceptual and methodological framework for studying inflammasome-mediated pathogenesis in gynecological diseases. It highlights the transformative capabilities of AI-assisted organoid systems in identifying biomarkers, predicting therapeutic responses, and enabling individualized immunomodulatory strategies—laying a foundation for next-generation translational research in reproductive immunology. To provide a comprehensive overview of recent advances in this field, we conducted a literature search primarily using the PubMed database, with keywords including “inflammasome,” “artificial intelligence,” “organoid,” and “gynecological inflammation,” as well as related terms. The review mainly focuses on experimental and methodological studies published within the past five years, aiming to reflect the most current developments in this rapidly evolving area.

## 1 Introduction

Gynecological diseases constitute a major and widespread risk to global public health; their aggregate burden is reported to surpass that of malaria and tuberculosis (1). Despite advances in medical studies, several obstacles persist in clinical care. Endometriosis is frequently diagnosed after prolonged delay, and there is an absence of reliable diagnostic biomarkers (2, 3). Treatment of ovarian cancer is often complicated by recurrences and drug resistance, and there is insufficient research on the immune environment of ovarian cancer (4, 5). Accordingly, further elucidation of the complex pathogenesis of gynecological diseases is crucial for optimizing treatment strategies. Inflammasomes are key multiprotein complexes of innate immunity that activate caspase-1 to induce pyroptosis by recognizing pathogen-associated molecular patterns (PAMPs) as well as damage-associated molecular patterns (DAMPs) (6, 7). Inflammasomes directly drive inflammation, encompassing both infectious and sterile inflammatory responses, thereby contributing to classic gynecological inflammatory diseases such as reproductive tract inflammation, endometritis, and oophoritis. Furthermore, alterations in the inflammatory microenvironment caused by inflammasome-driven inflammation can lead to pregnancy-related complications, including recurrent implantation failure, miscarriage, and preeclampsia. In the pathogenesis of these pregnancy-associated disorders, inflammasome-mediated disruption of inflammatory homeostasis lies at the core of the disease mechanism (8–11). As a central regulatory hub of innate immunity, the inflammasome is central to understanding these pathologies. Nevertheless, conventional models remain poorly suited to resolve the mechanisms governing its activation. Two-dimensional cell culture systems cannot reconstruct the complex cell interactions and signaling networks found in the three-dimensional microenvironment *in vivo*. Due to species differences, animal models also have limitations in accurately simulating the characteristics of human gynecological diseases, particularly in the regulation of estrogen-dependent inflammation. These issues pose significant challenges to the clinical translation of inflammasome-targeted drugs, creating what is known as the “translational bottleneck” (12–14).

Organoids, 3D cell clusters formed through the differentiation and self-organization of stem cells, could highly recapitulate the heterogeneity, structure, and physiological functions of their source tissues, providing an unprecedented “*in vitro* window” for studying gynecological diseases (15). The conversion of two-dimensional cell models into three-dimensional organoids is a process in which a three-dimensional microenvironment is constructed and developmental signals are regulated to enable the self-organization of monolayer cells. Seed cells are first expanded in a two-dimensional system and then embedded in a matrix scaffold to simulate the physical and chemical microenvironment *in vivo (*16); with the aid of growth factors and other means to precisely regulate signaling pathways, cells are driven to aggregate, establish polarity, and differentiate into multiple cell types; ultimately, they spontaneously form three-dimensional organoids with organ-like structures and functions (17). As three-dimensional cell aggregates, the construction of organoids mainly depends, in addition to cells, on customized culture media, specific growth factors and inhibitors, three-dimensional culture modes, and artificial scaffold materials. By adding key factors such as estrogen to regulate cells (18); by using Matrigel embedding, air-liquid interface, or suspension culture to construct a three-dimensional microenvironment; and by using materials such as Matrigel and synthetic hydrogels to provide physical support, cells ultimately self-assemble into functional organoid structures (19, 20). Compared with the *in vitro* culture of human tissues, organoids have broad sources, can be stably passaged and cultured for a long time, have a short construction cycle, and offer strong controllability; whereas human tissues, although closer to the real *in vivo* state, suffer from scarce sample sources, strong individual heterogeneity, short *in vitro* survival time, and ethical restrictions. Therefore, the construction of organoids is more suitable for standardized and high-throughput operations, providing ideas for AI-assisted research.

Organoid *in vitro* models and animal experiments can complement and cross-validate one another. However, organoid culture systems are inherently simplified, lacking, for example, a complete vascular network, an immune microenvironment, and systemic metabolic regulation (21). Conversely, animal models are subject to species differences and cannot achieve complete accuracy when simulating human gynecological diseases (22, 23). Both approaches have their own limitations. Therefore, organoids can be used independently for preliminary mechanistic exploration and drug screening, with key research findings subsequently validated through animal experiments, thereby more accurately reflecting human pathophysiological processes.

Artificial intelligence (AI) tools offer the necessary capabilities to process and analyze the massive, high-dimensional data generated by such high-fidelity models. Currently, AI is being used for the standardization and high-throughput construction of organoids (24), dynamic monitoring of inflammasome activation (25), and multi-omics integration to uncover the regulatory networks of inflammasomes in tumor microenvironments (26, 27).

The integration of inflammasome mechanism analysis, organoid model construction, and AI technology is rapidly becoming a new trend in precision research for gynecological diseases. AI has been used to conduct deep learning to target key genes in the NLRP3 pathway by profiling the transcriptome of endometrioid organoids (28) and track immune-tumor cell interactions in ovarian cancer organoids using microfluidic technology (29). This review systematically examines the pathogenic mechanisms of inflammasomes in gynecological diseases, the methods and technical bottlenecks of organoid model construction, and the enabling role of AI technology in the study of inflammasomes through multi-omics integration, image analysis, and experimental optimization. The review also looks forward to the future prospects of integrating these three approaches to the precise diagnosis and treatments of gynecological diseases. It aims to provide theoretical support and practical paths for related fields.

## 2 Inflammasomes

### 2.1 Overview of inflammasomes

The inflammasome is a multiprotein complex that detects pathogen-associated molecular patterns (PAMPs) and damage-associated molecular patterns (DAMPs) via pattern recognition receptors (PRRs), thereby modulating the activation of caspase-1.It also facilitates the maturation and secretion of pro-IL-1β and pro-IL-18, thereby contributing to the innate immune defense response (30, 31). It controls caspase-1-dependent programmed cell death. Under pathological conditions like inflammation and stress, this process induces cell death and drives inflammatory response activation (32). All in all, inflammasomes promote the release and maturation of various pro-inflammatory factors, thus contributing to inflammation and pyroptosis.It is worth distinguishing that pyroptosis is a caspase-dependent programmed cell death mode, characterized by the activation of inflammatory factors and the formation of cell membrane pores (33). On the other hand, extracellular vesicles (EVs) are phospholipid bilayer structures secreted by cells, which can transfer active substances and important mediators in the body and act as messengers for intercellular communication (34).

### 2.2 Structure and function of inflammasomes

The inflammasome comprises three components: sensor proteins, adaptor proteins, and effector proteases. Sensor proteins are the primary basis for the classification of inflammasome subtypes and can be divided into the PYHIN and NLR families (11, 35). These proteins detect and transmit signals induced by self or non-self stimuli. PYHIN inflammasomes primarily detect double-stranded DNA. Their function is critical in viral infection-related gynecological diseases. IFI16 is a PYHIN protein. It increases PD-L1 expression via the STING-TBK1-NF-κB pathway, thereby accelerating the progression of cervical cancer (36). Members of the NLR family are stimulated by pathogen proteins and metabolites, contributing to the development of gynecological inflammatory diseases (37). Exploring the targets of sensor proteins in both families may aid in developing targeted therapies for cervical cancer.

The adaptor protein ASC, an apoptosis-associated speck-like protein containing caspase activation and recruitment domains, serves as a molecular bridge connecting sensor proteins with effector proteases. Upon receiving signals from sensor proteins, ASC activates effector proteases.

Effector proteases are the final components of the inflammasome and are responsible for promoting the maturation of IL-1β and IL-18 precursors. Additionally, effector proteases cleave PRO-GSDMD to form GSDMD. GSDMD can oligomerize to form perforations in the target cell membrane, promoting the active efflux of IL-1β and IL-18 from the target cells, thereby initiating inflammation (38).

### 2.3 The role of inflammasomes in gynecological diseases

Inflammasomes can either directly or indirectly help to release many pro-inflammatory chemicals, trigger something called pyroptosis, and recruit immune cells. They play a really important role in the start and development of gynaecological disorders, like endometriosis, ovarian cancer, polycystic ovary syndrome and miscarriage (11, 32).

#### 2.3.1 Inflammasomes and endometriosis

Endometriosis is a chronic gynecological condition affecting approximately 5–10% of women of reproductive age worldwide, characterized by the ectopic implantation and growth of endometrial tissue outside the uterine cavity (39, 40). While endometriosis is considered a benign gynecological disease, it significantly impacts patients’ lives. The severity of endometriosis correlates with the viability index and migration activity of ectopic human endometrial stromal cells (hESC). Elevated expression of the NLRP3 inflammasome enhances the viability and migration of human endometrial stromal cells (hESCs) via the NLRP3/caspase-1/IL-1β signaling pathway, thereby driving disease progression (41, 42).

**Figure 1:**
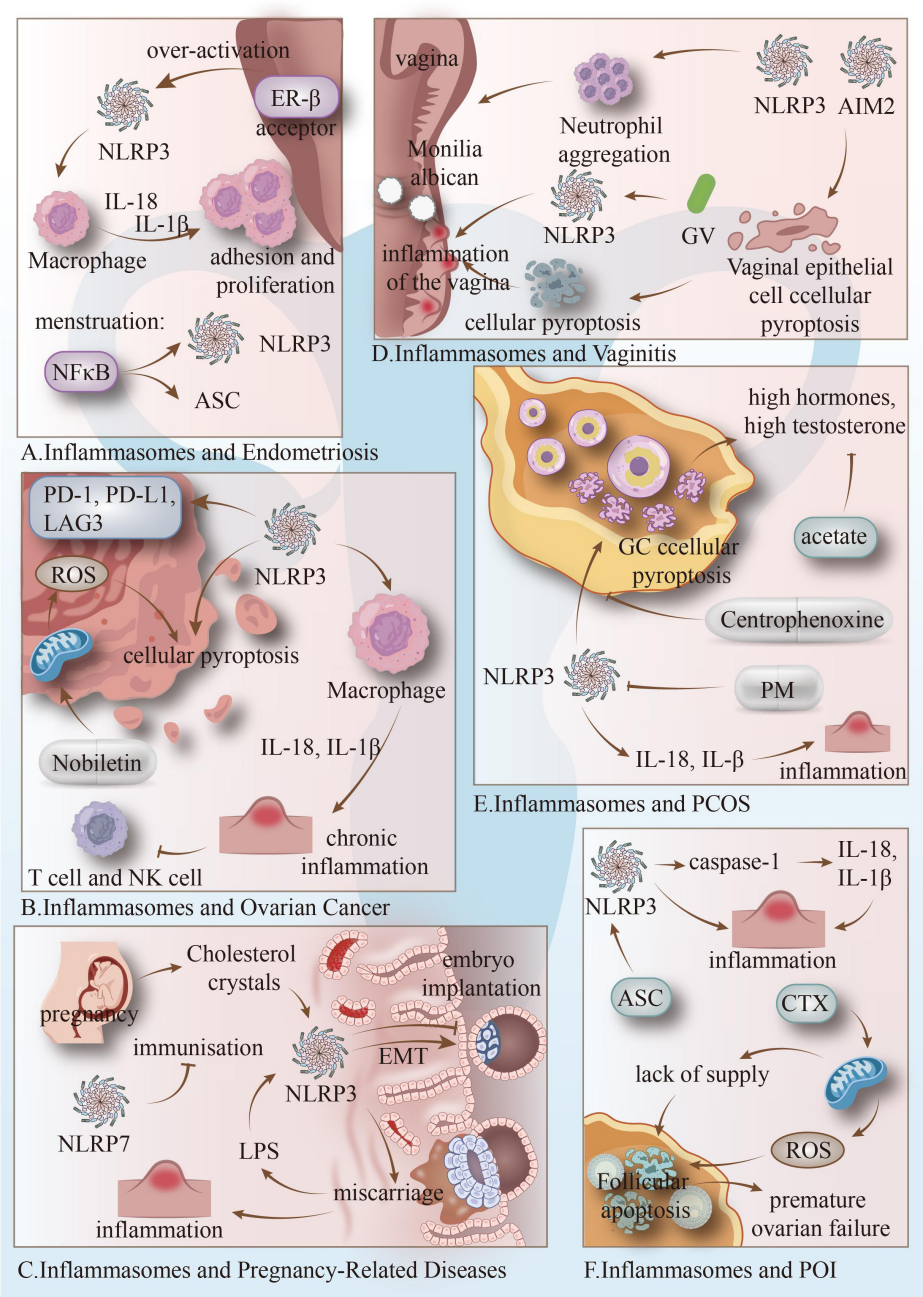
**(A)** In endometriosis, NLRP3 is activated via the NLRP3/caspase-1/IL-1β signalling pathway, enhancing the viability and migratory capacity of endometrial stromal cells and driving disease progression; elevated ER-β and NF-κB activation further promote this inflammasome activation. **(B)** In ovarian cancer, NLRP3 inflammasome upregulates PD-1/PD-L1/LAG3 expression to mediate immune evasion while inducing tumor cell pyroptosis, thereby influencing disease progression by regulating the inflammatory microenvironment. **(C)** In pregnancy-related disorders, NLRP3 inflammasome promotes embryo implantation by regulating endometrial receptivity and EMT processes. Its abnormal activation induces uterine inflammation, accelerates placental aging, and increases risks of miscarriage and preterm premature rupture of membranes. **(D)** In vaginitis, NLRP3 inflammasome activation mediates vaginal mucosal inflammation and epithelial cell pyroptosis. AIM2 deficiency exacerbates pathological damage, while GSDMD cleavage participates in anti-Candida infection defense, collectively regulating disease progression. **(E)** In polycystic ovary syndrome, NLRP3 overexpression induces granulosa cell pyroptosis, releasing IL-18/IL-1β to trigger chronic ovarian inflammation and fibrosis, worsening insulin resistance and follicular dysfunction; targeted inhibition of this pathway alleviates symptoms. **(F)** In premature ovarian failure, upregulation of the NLRP3/caspase-1/IL-1β pathway accelerates ovarian granulosa cell apoptosis and follicular depletion, mediating disease onset; downregulating inflammasome expression improves the ovarian microenvironment and delays follicular loss.

Endometriosis is estrogen-dependent. Elevated levels of estrogen receptor β (ER-β) in pathological tissues lead to excessive activation of the NLRP3 inflammasome. This inflammasome activation increases the production of IL-18 and IL-1β, which promote cell adhesion and proliferation (43). Symptoms of endometriosis often correlate with the menstrual cycle. During menstruation, NF-κB activation significantly increases NLRP3 and ASC levels, triggering an inflammatory response similar to that seen in menstrual endometrium (44). Inhibiting the ROS/NLRP3 inflammasome signaling pathway alleviated endometrial fibrosis (45). While targeting inflammasomes can induce pyroptosis in ectopic endometrial cells, excessive inflammasome activation may also cause tissue damage (46) (**Figure 1A**).

#### 2.3.2 Inflammasomes and ovarian cancer

In ovarian cancer, increased NLRP3 inflammasome levels promote the expression of immune checkpoints such as PD-1, PD-L1, and LAG3 in cancer cells, facilitating immune escape. As a result, high NLRP3 levels are often associated with poor prognosis in ovarian cancer (47). Activation of inflammasomes induces the production of IL-18 and IL-1β, promoting chronic inflammation, inhibiting NK and T-cell surveillance, and facilitating cancer progression (48). However, some evidence suggests that inflammasomes may inhibit cancer development. For instance, nobiletin induces ROS release, activating inflammasomes and promoting pyroptosis in ovarian cancer cells (49, 50). In mouse models, depletion of NLRP3 inflammasomes in macrophages significantly increased cancer cell growth and metastasis, possibly due to reduced macrophage phagocytosis and fewer cytotoxic CD8+ T cells (51). Inflammasome-mediated pyroptosis can cause cell membrane perforation, leading to cell swelling and inhibition of ovarian cancer development (52). Cisplatin resistance in ovarian cancer cells may be associated with the suppression of NLRP3 activation by estrogen-related receptor α (ERRα), which consequently inhibits pyroptosis (53). However, the role of inflammasomes in ovarian cancer remains controversial, and further studies are needed (54) (**Figure 1B**).

#### 2.3.3 Inflammasomes and pregnancy-related diseases

Pregnancy is a intricate and lengthy process, encompassing stages from fertilization to embryo development and birth. Pregnancy is maintained by a unique immune state. Its success depends on a stable inflammatory environment regulated by inflammasomes. Under pathological inflammatory conditions, however, excessive or insufficient inflammasome activity disrupts this immune equilibrium. This disruption leads to adverse pregnancy outcomes and complications, such as recurrent implantation failure (RIF), spontaneous miscarriage, preterm birth, and preeclampsia. Consequently, these pregnancy-related disorders form an essential part of any discussion on inflammasome-associated gynecological inflammation. They demonstrate how dysregulation of inflammasome-mediated inflammation drives pregnancy complications and adverse outcomes. NLRP3 is abundantly expressed in the human endometrium during the mid-proliferative and mid-secretory phases, promoting the transcriptional upregulation of NLRP3 inflammasome-related genes by binding to ER-β. Moreover, NLRP3 facilitates embryo implantation through inflammasome-dependent and independent pathways, enhancing EMT in Ishikawa (IK) cells, thus making the endometrium more receptive to embryo adhesion and implantation (55).

However, dysregulated activation of the inflammasome can impair embryo implantation, resulting in recurrent implantation failure and spontaneous miscarriage (56). During pregnancy, cholesterol crystals can accumulate in the decidua, activating the NLRP3 inflammasome and leading to decidual inflammation, which increases the risk of miscarriage (57).

In the case of recurrent miscarriage, intestinal leakage and lipopolysaccharide (LPS) may enter the bloodstream, causing excessive activation of the NLRP3 inflammasome in the endometrium (56). Additionally, during spontaneous abortion, NLRP3 inflammasome activation adversely affects endometrial receptivity by promoting inflammatory responses in the placenta and accelerating placental aging (58, 59). The NLRP7 inflammasome, a member of the NLR family, is also a cause of recurrent complete hydatidiform mole. NLRP7 inflammasomes promote the transformation of recurrent complete hydatidiform mole into gestational choriocarcinoma by enhancing the proliferation and three-dimensional structure construction of gestational choriocarcinoma cells in an inflammasome-independent manner, while simultaneously inhibiting the maternal immune response and accelerating carcinogenesis (60). Additionally, the NLRC4 inflammasome has been shown to contribute to term premature rupture of membranes (TPROM) (61), and may also be associated with NLRP3 inflammasome overactivation in preterm membrane rupture (PROM) (62). NLRP3 inactivation in the human uterus contributes to its resting state, and promoting its activation can trigger parturition (63, 64) (**Figure 1C**).

#### 2.3.4 Inflammasomes and vaginitis

Vaginitis is a common gynecological disease with a variety of causes. In persistent fungal vaginitis caused by Candida albicans, activation of the NLRP3 inflammasome and lack of AIM2 inflammasomes can lead to excessive neutrophil aggregation and pyroptosis of vaginal epithelial cells, resulting in damage to the vaginal epithelial mucosa and prolonging the disease (65, 66). Activation of the NLRP3 inflammasome also serves as a pivotal driver of mucosal inflammatory responses, predominantly involving neutrophils, in recurrent vaginal Candida infection (67). Furthermore, inflammasome signaling can have a protective effect against Candida albicansinfections by mediating the cleavage of gasdermin D (GSDMD), a key mediator in the inflammasome pathway (68). It has also been shown that Gardnerella vaginalis can induce bacterial vaginitis by activating NLRP3 inflammasomes and triggering pyroptosis (69) (**Figure 1D**).

#### 2.3.5 Inflammasomes and polycystic ovary syndrome

Polycystic ovary syndrome (PCOS) is a multifactorial endocrine and metabolic disorder affecting nearly 15% of women of reproductive age worldwide, imposing a substantial economic and health burden on affected individuals (70–73). The clinical features of PCOS encompass hyperandrogenism, elevated testosterone levels, hirsutism, insulin resistance, and infertility (73). In PCOS, ovarian granulosa cells (GC) surround oocytes to form follicles, providing a suitable environment for follicle maturation. However, elevated levels of NLRP3 inflammasomes induce granulosa cell pyroptosis, accelerating the onset and progression of the disease (74). Chronic ovarian inflammation, driven by IL-18 and IL-1β released upon inflammasome activation, also promotes ovarian fibrosis and follicular dysfunction, further exacerbating the disease (75). This effect is particularly pronounced in obese patients (75, 76). Reducing inflammasome expression can alleviate heart and kidney diseases associated with PCOS (77). Notably, plumbagin, a therapeutic compound, can inhibit inflammasome-dependent granulosa cell pyroptosis, thus providing a potential treatment for PCOS (78). Moreover, metformin and pioglitazone complex preparations have been shown to alleviate psychological distress in patients with PCOS through the suppression of NLRP3 inflammasome activation (79), whereas acetate has been reported to alleviate PCOS-related complications by reducing androgen excess and attenuating NF-κB/NLRP3 immunoreactivity (80) (**Figure 1E**).

#### 2.3.6 Inflammasomes and premature ovarian failure

Premature ovarian failure (POI) is a major cause of female infertility and endocrine dysfunction, leading to early menopause and reduced fertility (81). The clinical symptoms of POI include impaired ovarian function, decreased fertility, and premature menopause (82). Premature depletion of ovarian follicles is a major cause of POI, and the inflammatory microenvironment accelerates follicular apoptosis (83). Increased expression of inflammatory markers, including NLRP3 inflammasome, caspase-1, and IL-1β, is observed in the granulosa cells of POI patients. In a POI mouse model, inhibition of NLRP3 expression alleviated follicular apoptosis and ovarian aging, thereby improving ovarian function (84). Traditional Chinese medicine treatments, such as moxibustion, have been demonstrated to downregulate the expression of NLRP3 and ASC, alleviate granulosa cell apoptosis, and improve the ovarian microenvironment, thus promoting follicle growth and delaying follicle depletion (85). Furthermore, the transplantation of placental mesenchymal stem cells (PMSCs) improved ovarian function in POI rats by inhibiting NLRP3 inflammasome activation (86) (**Figure 1F**).

#### 2.3.7 Inflammasomes and cervical cancer

Cervical cancer continues to pose a significant global health burden for women, with high morbidity and mortality rates due to inadequate screening programs (87). The primary etiological factor of cervical cancer is persistent infection with high-risk human papillomavirus (HPV) types, particularly HPV-16 and HPV-18 (88). HPV-16 not only activates AIM2 and IFI16 inflammasomes through its own DNA (89), but also indirectly activates NLRP3 through HPV oncoproteins E6 and E7 (90–92). Both of these mechanisms promote pyroptosis. However, HPV E7 can suppress pyroptosis and evade immune surveillance by recruiting the E3 ubiquitin ligase TRIM21 to mediate the ubiquitination and degradation of the IFI16 inflammasome (93). Reduced levels of IL-1β and IL-18 mRNA in cervical tissue have been identified as risk factors for the progression of CIN to cancer, suggesting that these cytokines could serve as potential biomarkers for risk stratification (94). Additionally, single nucleotide polymorphisms (SNPs) in the NLRP3 gene, particularly rs10754558, have been identified as significant risk factors for cervical cancer (95).

#### 2.3.8 Inflammasomes and pelvic inflammatory disease

Pelvic inflammatory disease (PID) is an infectious inflammation of the female upper reproductive tract, most commonly induced by sexually transmitted pathogens such as Neisseria gonorrhoeae and Chlamydia trachomatis, as well as by microorganisms associated with bacterial vaginosis (96). Studies have shown that Chlamydia trachomatis can effectively activate the NLRP3/caspase-1/IL-1β pathway upon infecting cervical epithelial cells (97). Caspase-1 causes the fragmentation of the host cell Golgi, which provides lipids for Chlamydia replication, leading to prolonged infection (98). Chronic release of downstream cytokines like IL-1β and IL-18 recruits immune cells, aggravating local tissue damage, causing tubal edema, fibrosis, and adhesion, and ultimately leading to severe sequelae (99) (**Tables 1**, **2**).

**Table 1:** Panorama of the integrated application of organoid models and artificial intelligence in gynecological disease research.

Disease	Organoid model application	AI technology assistance	References
Endometriosis	Endometriosis organoids can stably reconstruct the inflammatory pathways and estrogen response in the lesions, used for mechanistic studies and drug screening.	Analyzing the organoid transcriptome features to identify key genes in the NLRP3 pathway, aiding in mechanistic analysis; screening diagnostic biomarkers.	(28, 100–102)
Ovarian Cancer	Ovarian cancer organoids (PDO) retain the genomic variations of the primary tumor and immune microenvironment features, used for drug sensitivity testing and high-throughput screening.	Widely applied in imaging genomics, whole-slide imaging (WSI), etc., to assist in ovarian cancer diagnosis, distinguishing benign and malignant cases, and prognosis evaluation.	(103–107)
Pregnancy-Related Diseases (Including Embryo Implantation, Miscarriage, Hydatidiform Mole, etc.)	Endometrial Organoid Models for Studying Influencing Factors in Complex Embryo Implantation, Including the Inflammatory Microenvironment.	Constructing live birth prediction models after fresh embryo transfer in PCOS patients; identifying hydatidiform mole (HM) lesions under the microscope to assist in diagnosis.	(108–110)
Vaginitis	Vaginal epithelial organoids (VEO) co-cultured with CD8 T cells to study the influencing factors of resident memory T cell (TRM) formation.	Used for microbiome evaluation and biomarker screening in bacterial vaginosis (BV); used for microscopic image identification, assisting in the diagnosis of fungal vaginitis.	(111–113)
Polycystic Ovary Syndrome	Endometrial epithelial organoids (EEOs) derived from PCOS patients are used to study PCOS-related endometrial epithelial dysfunction.	Assisting in PCOS diagnosis and early intervention, ultrasound image differentiation; discovering potential biomarkers and pathogenesis.	(114–116)
Cervical Cancer	Cervical cancer organoids retain genetic heterogeneity, simulating HPV infection and tumorigenesis, used for personalized modeling.	Imaging analysis (such as MRI radiomics models) to assist cervical cancer diagnosis, predicting chemotherapy survival.	(117–119)

**Table 2:** The mechanism of inflammatory bodies in gynecological inflammatory diseases.

Family	Classification	Activation factors	Effector molecules	Mechanism in gynecological inflammatory diseases	References
NOD-like receptor family (NLR)	NLRP1	Pathogen-associated molecular patterns (PAMPs) and damage-associated molecular patterns (DAMPs)	IL-1β , IL-18, GSDMD	Activates nociceptors (TRPA1), leading to widespread pain associated with endometriosis.	(120, 121)
	NLRP3			Amplifies local inflammation, inhibits macrophage and CD8+ T-cell activity, promotes immune escape of tumor cells.	(74, 75)
	NLRP7			Suppresses maternal immune response, while promoting recurrence of complete hydatidiform mole (HM) to gestational trophoblastic disease.	(60)
	NLRC4			Suppresses inflammation and NLRP3 inflammasome, inhibits the progression of vulvovaginal candidiasis.	(122)
Pyrin and HIN domain family (PYHIN)	AIM2	Viruses, especially double-stranded DNA viruses		Promotes neutrophil aggregation at inflammatory sites, prolongs the course of gynecological inflammation.	(53, 65, 66)
	IFI16			Upregulates PD-L1 via the STING-TBK1-NF-κB pathway, promoting the progression of cervical cancer.	(36)

## 3 Establishment of organoids and their application in gynecological diseases

### 3.1 Overview of organoids

The theory of evolutionary conservation of biological principles has made animal models the primary approach to studying the basic mechanisms of development and disease, but even basic biological processes may differ significantly across species (14). However, human-specific processes such as brain development, metabolism, and drug efficacy testing cannot be effectively modeled in animals (13). Organoids, which mimic the physiological and pathological environment of organs, offer a promising platform for such research (12, 123).

Organoids refer to miniature, three-dimensional (3D) cell clusters formed from induced pluripotent stem cells (iPSCs), adult stem cells (ASCs), or patient-derived cells. Self-organising and differentiating into functional cell types, these organoids closely resemble the structure and function of the organs they represent (15, 124). Organoids have vast potential in regenerative medicine, precision medicine and drug research (124). They are being increasingly used in various disease modeling studies (125–127).

### 3.2 Construction of organoids for gynecological diseases

#### 3.2.1 Establishment of tumor immune microenvironment

The tumor microenvironment (TME) is defined as the complex and diverse environment surrounding tumor cells, comprising various immune cells, tissue cells, adipocytes, and extracellular matrix components. It serves a crucial role by supporting tumor growth and survival (128–130). The immune-related components within TME are collectively termed the tumor immune microenvironment (TIME), which focuses on the immune regulation mechanisms that affect tumor progression (131–133).

Studies have shown that complex interactions between tumour cells, immune cells and local stroma are associated with lymphatic metastasis in cervical cancer (134). For example, tumor-associated macrophage M2 (TAM) in the TME promote the expression of PD-L1 in cervical cancer cells through the PI3K/AKT pathway, enhancing their metastatic and invasive abilities, which affects tumor progression (135). Similarly, metastasis in ovarian cancer is linked to FOXP3+ regulatory T cells, which suppress the growth of effector T cells by producing the immune checkpoint molecule PD-1. This activation of the PD-1/PD-L1 pathway has an immunosuppressive effect on the tumour microenvironment, promoting immune evasion and metastasis (136–138).

Constructing an accurate TIME model is crucial for gynecological disease research, and two main methods are employed: reconstruction models and non-reconstruction models (139). In the reconstructed model, pure tumour cell organoids are co-cultured with exogenous immune cells to mimic the morphological and genetic characteristics of tumours, though these organoids lack endogenous immune infiltration. In the non-reconstructed model, it can preserve tumor cells and their endogenous immune cells, primarily cultivated through two methods:air-liquid interface (ALI) culture and 3D microfluidic culture systems (PDOTS/MDOTS).

ALI culture involves physically dividing patient-derived tumor tissue into small fragments and culturing them in a chamber where one side is exposed to air while the other is immersed in liquid culture medium (29, 125, 140). This method preserves the TME structures, including functional tumor-infiltrating lymphocytes (TILs), and is particularly useful for modeling TIME *in vitro*.

PDOTS/MDOTS involves embedding tumor spheres from patient-derived tissues in a 3D microfluidic device with collagen for co-culture (29, 140). This approach also preserves multiple functional immune cell populations and enables more precise control of the microenvironment (29, 141). Vikram et al. investigated the influence of neutrophils on the initiation of collective 3D invasion by ovarian tumor cells by designing a novel, microfluidically integrated 3D-TIME-on-chip device. They demonstrated that neutrophil extracellular traps play a crucial role in mediating collective invasion of human ovarian cancer cells from a clustered state (142).

#### 3.2.2 Initial cell population for organoid construction in gynecological diseases

Organoid cultures can be generated from induced pluripotent stem cells (iPSCs), adult stem cells (ASCs), or patient-derived cells (143, 144). Naive cells can self-organize and form tissue-specific organoids when provided with appropriate 3D substrates and specific signaling instructions (144–147). Different cell types follow distinct developmental pathways (127, 144). Organoids derived from iPSCs resemble fetal tissues and are primarily used to understand diseases associated with developmental defects. Organoids derived from ASCs highly recapitulate the homeostasis and regenerative capacity of their source tissues, making them widely employed for disease modeling (148–150).

##### 3.2.2.1 Induced pluripotent stem cells

**Figure 2:**
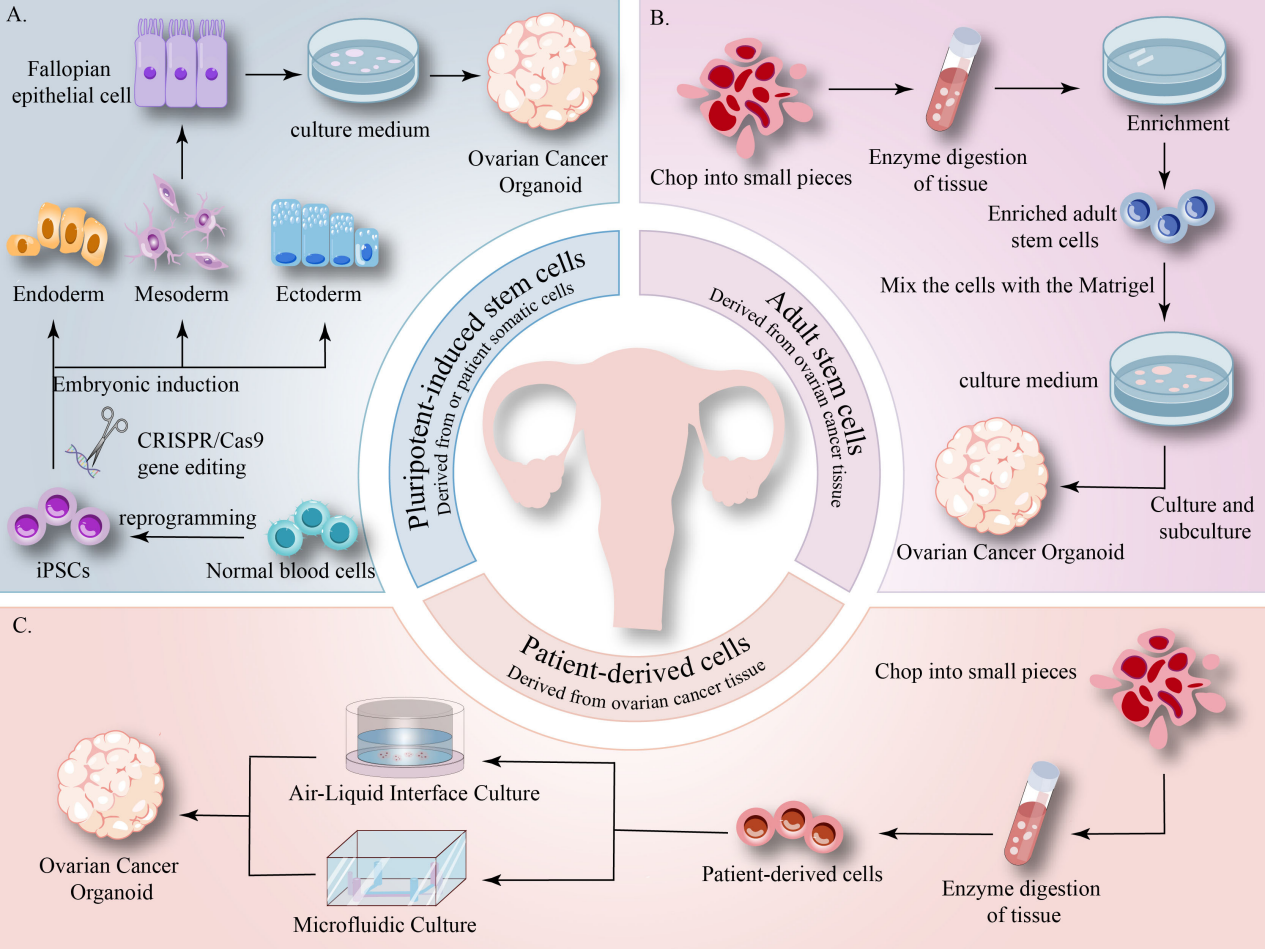
**(A)** iPSCs possess self-renewal and tri-germ layer differentiation potential, derived from reprogramming patient somatic cells, providing a disease-relevant genetic background for constructing organoids of gynecological diseases. **(B)** ASCs reside in specific differentiated tissues, exhibiting robust proliferation and differentiation capabilities. Organoids derived from ASCs closely resemble their tissue of origin, enabling rapid construction of models that accurately mimic primary tumors. **(C)** Patient-derived cells primarily consist of primary tumor cells, which can be cultured into organoids without reprogramming. These organoids retain key characteristics of the original tumor, aiding preclinical research.

iPSCs are one of the primary sources for organoid formation, characterized by their self-renewal capacity and ability to differentiate into specialized cell types derived from the three germ layers: ectoderm, endoderm, and mesoderm (151, 152). Gynecological target cells can be obtained by reprogramming patient-derived somatic cells into iPSCs, followed by expansion and differentiation. These somatic cells carry disease-causing mutations or genetic risk factors, and the resulting iPSCs possess a disease-associated genetic background (103, 153). iPSCs derived from BRCA1 mutation patients can differentiate into fallopian tube organoids, which can be modeled to simulate high-grade serous ovarian cancer (154, 155). iPSC-derived organoids can provide insights into early organogenesis and diseases related to developmental defects (12, 148) (**Figure 2A**).

##### 3.2.2.2 Adult stem cells

ASCs are undifferentiated cells present in specific differentiated tissues that possess high proliferative potential as well as the ability to differentiate into various cell types. Organoids derived from ASCs closely r esemble the tissue of origin and retain its characteristics (156–158). In cancer research, ASCs-derived organoids maintain the genomic integrity and TME of the original tumor (158). Moreover, AdSCs can be directly obtained from regenerating human adult tissues, with their derived organoids mature more closely to adult tissues compared to iPSC-derived organoids, enabling quicker establishment of tumor organoids that accurately simulate original tissues (12, 151) (**Figure 2B**).

##### 3.2.2.3 Patient-derived cells

Patient-derived cells primarily refer to primary tumor cells obtained directly from the patient’s tumor tissue. Culturing organoids from these cells requires no reprogramming, and the resulting organoids highly preserve heterogeneity while maintaining the key phenotypic and genetic characteristics of the source tumor (159). Patient-derived tumor organoids serve as a vital preclinical model for drug sensitivity prediction and cancer progression research (151, 160) (**Figure 2C**).

#### 3.2.3 Application of organoid construction in gynecological diseases

Although organoid technology is still in its early phases, it holds tremendous potential in regenerative medicine, drug research, and precision medicine (124). Several gynecological disease organoid models, such as those for ovarian cancer and cervical cancer, have already been established for studying the pathogenesis of gynecological diseases (161).

##### 3.2.3.1 Ovarian cancer

Ovarian cancer (OC) is the deadliest malignant gynecological tumor, ranking eighth in cancer incidence and mortality among women worldwide (162). It is highly heterogeneous, with different subtypes exhibiting distinct clinical and molecular characteristics (163, 164). Organoids derived from ovarian cancer tissues, such as fallopian tube epithelium, ovarian cancer *in situ*, and ascitic ovarian cancer tissues, have been successfully constructed for disease modeling (164). The collected samples are mechanically and enzymatically digested to form a cell suspension, then plated using basement membrane extract, and supported with a medium supplemented with additives specifically for culturing ovarian cancer cells (165, 166).

Ovarian cancer (OC) organoids, as an emerging preclinical *in vitro* tool, are crucial for studying pathogenesis (167). The majority of OC cases currently exhibit an epithelial phenotype, known as epithelial ovarian cancer (EOC) (168). It has been reported that organoids derived from EOC can recapitulate the disease and the original tumor phenotype, replicate the genomic and mutational profile of the primary tumor, and have been successfully engineered, providing a foundation for mechanistic studies (169). Additionally, compared to animal models, ovarian cancer organoids possess advantages such as clonality, the potential for high-throughput screening, and lower costs, which simplifies the complex processes in the preclinical drug development phase, making them suitable for drug screening and development (167, 170).

##### 3.2.3.2 Endometriosis

Endometriosis is an estrogen-dependent chronic gynecological condition characterized by the growth of endometrial-like tissue outside the uterus. The resulting chronic pelvic pain and infertility severely impact the well-being of women of reproductive age (3). Meanwhile, it also significantly raises the incidence of OC (171). The application of organoids in modeling this disease has garnered significant interest. For instance, placental protein(Gda) is a glycoprotein produced by the endometrium that mediates several biological processes involved in human reproduction and maternal-fetal immunity. Its gene expression and modification varying throughout the menstrual cycle (172). At the same time, Gda also changes significantly during the menstrual cycle of female patients with Ems (173). Alice et al. found that endometrioid ovarian cancer organoids (EndORG) were able to generalize this specific expression pattern (174). Jiang Research also found that human endometrial organoids (hEO) can summarize the human menstrual cycle dynamics and respond to estrogen treatment (100). Both endometriotic tissue and normal endometrial tissue undergo cyclical changes in response to hormonal fluctuations, but the former exhibits invasive and metastatic properties, whereas the latter does not. Researchers have successfully established endometrial epithelial organoids and endometriotic epithelial organoids. They found that, compared to *in situ* endometrial epithelial organoids, most endometriotic epithelial organoids took longer to establish, grew more slowly, and had a slightly lower success rate. This may be because fibrosis of the endometrioma wall reduces the proportion of glandular epithelial cells in the ectopic tissue, thereby increasing the difficulty of establishing ectopic endometrial epithelial organoids (175), whereas an appropriate level of estrogen helps promote the establishment of ovarian endometriosis (OE) organoids (101). It can be concluded that the *in vitro* modeling of Ems by organoids can well summarize the disease, including the original *in vivo* characteristics. It also provides an excellent experimental model for drug intervention and mechanism research.

##### 3.2.3.3 Cervical cancer

Cervical cancer represents one of the most prevalent malignant tumors among women worldwide (176). It includes several histological types, such as squamous cell carcinoma (SCC), cervical adenocarcinoma (CA), and adenosquamous carcinoma (177). Due to the low detection rate of early-stage CA, its invasiveness, high recurrence rate, and mortality rate pose significant clinical challenges (177). Moreover, the current research on CA lacks sufficient preclinical models, while organoids have the ability to mimic the structure and function of patient tumour tissue, providing accurate and individualised *in vitro* platforms for disease modeling (177). CA organoids are typically established by taking biopsy tissue, dissociating and separating the cells with collagenase, embedding them in Matrigel matrix, and culturing them in organoid medium (178). These organoids retain host genetic and genomic heterogeneity (117). The development of cervical cancer is significantly influenced by HPV infection. It primarily infects the stratified squamous epithelial cells of the mucosa, where it replicates and proliferates. Epithelial cells with glandular components in the cervix may provide a microenvironment conducive to HPV replication (177). Therefore, CA organoids can be used to simulate the process of HPV-induced tumors. In reality, however, the successful establishment of CA organoids is rare, which may be a finding for future research (117, 177).

##### 3.2.3.4 Polycystic ovary syndrome

PCOS is a complex disorder of the endocrine and metabolic systems. Its primary clinical features include elevated levels of androgens, polycystic ovaries, and impaired ovulation, leading to reproductive dysfunction, chronic low-grade inflammation, and abnormal metabolism like insulin resistance (179). Teerawat et al. investigated the influence of increased androgen concentrations on the endometrium in PCOS patients by establishing scaffold-free multicellular endometrial organoids. They found that excess androgens directly affect endometrial cells (180). Lu et al. also observed the same adverse effects in human trophoblast organoids as those seen in animal models treated with androgen stimulation. Furthermore, these adverse effects could be prevented by blocking androgen receptors (181). A recent study, using endometrial organoids (EEO), identified for the first time a clear link between the GPX4-TGF-β1/Smad2/3 signaling pathway and fibrosis in the endometrial epithelial cells of PCOS, providing a direction for therapeutic strategies to improve PCOS-related endometrial function (114). Thus, the establishment of PCOS organoids not only facilitates the exploration of its pathogenesis but also provides a foundation for therapeutic strategies that reduce the impact of this disease on pregnancy, as well as on women’s overall health (182).

## 4 Artificial intelligence

### 4.1 Current status of artificial intelligence in modern medicine

Artificial Intelligence (AI) can be defined as the use of computer systems to imitate intelligent behaviour with minimal human assistance (104). With continuous improvements in algorithmic performance and the rapid accumulation of biomedical data, AI has progressively expanded from predominantly macroscopic applications in clinical imaging–based diagnosis to data interpretation at the cellular and molecular scales, and its potential value in mechanistic research has become increasingly evident. In medical research, AI was initially applied to imaging data analysis; deep learning–based models have demonstrated strong performance in automatically identifying abnormal structures in CT, MRI, and histopathological sections (105, 183–188). These advances have also opened new avenues for applications in gynecological diseases (115, 118, 189–196). However, in recent years, the focus of AI applications has gradually shifted from macroscopic imaging diagnosis toward microscopy-scale biological research.

In high-content imaging (HCI)–based analyses, AI enables automated identification, classification, and quantification of large numbers of cellular and subcellular structures, thereby supporting systematic profiling of complex cellular phenotypes. Using deep learning models, AI can detect subtle morphological differences, changes in intracellular protein distributions, and spatial patterns of subcellular structures. This allows researchers to extract high-dimensional features from large-scale image datasets and to further analyze dynamic cellular responses under different experimental conditions (197, 198). At single-cell resolution, deep learning–assisted microscopic image analysis can capture heterogeneous features within cell populations. This is particularly important for investigating biological events such as dynamic inflammatory responses, immune cell activation, and signal transduction processes, because heterogeneity at the single-cell level is often masked by population-averaged data (199, 200). For example, in inflammasome research, ASC speck formation is a morphological hallmark of activation and can be detected by fluorescence microscopy, flow cytometry, or HCI. AI can not only accurately count these specks but also analyze their spatial distribution and temporal dynamics, enabling quantitative determination of inflammasome activation states (201, 202).

Meanwhile, AI integrates multi-omics data to identify individualized disease risks, pathogenesis, and therapeutic targets (203, 204). High-throughput screening of potential biomarkers through multi-omics integration (116, 205). AI-driven virtual screening, big data mining, and molecular generation algorithms have significantly shortened the development cycle and reduced costs in drug research (206–208). Furthermore, AI-assisted surgical systems, capable of achieving millimeter-level precision in complex surgeries, are becoming increasingly common, significantly enhancing the safety and efficiency of gynecological procedures (209–213). AI also assists novice doctors in performing professional examinations with the accuracy of experienced specialists (214). In addition, AI can integrate multi-source data—such as electronic medical records, pathology reports, and laboratory testing results—to support molecular characterization and longitudinal monitoring of gynecologic diseases (215–218).

Overall, the application of AI in medicine has evolved from macroscopic imaging analysis toward microscopic structural recognition, single-cell phenotypic profiling, and molecular-level data integration. This transition has positioned AI not only as a clinical decision-support tool but also as an increasingly important research approach for interrogating inflammation-related molecular pathways during tumor initiation and progression, thereby providing a technical foundation for systematic investigation of key mechanisms—such as inflammasomes—in organoid models.

### 4.2 Challenges and application scenarios of AI-assisted organoid research

Traditional organoid culture systems require the precise simulation of the *in vivo* microenvironment, involving media, extracellular matrix, growth factors, and other components. However, the culture conditions for different types of organs vary greatly, making it challenging to standardize (13, 219, 220). Additionally, traditional culture methods make high-throughput screening difficult, limiting the scale of sample research and drug development (13, 221). Organoid culture thus often relies on trial-and-error methods and expert knowledge, which can be inefficient and inconsistent, despite significant successes in growing organoids.While traditional animal models can fully replicate systemic physiological processes *in vivo* and support long-term *in vivo* observation and studies of overall mechanisms, they lack sufficient accuracy in simulating complex conditions such as human gynecological diseases due to species differences. Additionally, they suffer from drawbacks such as long study durations, high costs, strict ethical constraints, and poor experimental reproducibility (222, 223). In contrast, AI-assisted organoid culture can optimize construction strategies by accurately screening matrix materials, optimizing cell culture conditions, and dynamically monitoring culture conditions, cell behavior, and organoid development in real-time (24, 25, 224). It can efficiently process multi-scale images and multi-omics data, enhancing disease modeling, drug screening, immune checkpoint blockade simulations, and other applications, thus accelerating organoid research development (25, 221, 225–227). AI algorithms can analyse data from multiple sources to predict how stem cells will differentiate into specific cell types. This promotes the generation of more complex and precise organoids. This technology has shown potential not only in kidney, retina, and other organoid models (228–230), but also in gynecological-related organoid studies. For example, AI technology can integrate multi-platform metabolomics data to accurately detect intrauterine growth restriction (231, 232). By combining with natural language processing (NLP), AI can further explore and understand the function and regulatory mechanisms of endometriosis-related genes (231, 233). Additionally, AI can be employed to model placental development and assess placental susceptibility to emerging pathogens (234). Although AI-assisted organoid research holds significant value, its *in vitro* culture systems lack a complete *in vivo* microenvironment, making it difficult to simulate complex pathophysiological processes. Furthermore, since culture protocols have not yet been fully standardized and AI models rely on *in vitro* data, this can easily lead to discrepancies between predictive results and actual *in vivo* conditions (235). Therefore, by utilizing animal models to provide *in vivo* physiological references and validate the authenticity of organoid functions, AI models can be trained and validated against benchmarks that more closely resemble human diseases, thereby effectively enhancing the reliability of predictions and their clinical translation value.

Additionally, AI has a significant impact on drug research and prediction using organoids. AI technology can assist organ-on-a-chip (OoC) models in simulating the physiological activities and functions of human organs, thereby avoiding the ethical concerns of animal welfare and other drawbacks associated with traditional drug development models (236). In organoid research, AI can also analyze multi-omics data to deeply explore disease mechanisms and drug responses; by analyzing time-series data from organoids, AI can simulate organoid development in both normal and diseased states (237), revealing organoid-specific developmental functions and pathways (238). Furthermore, AI can be used to understand different immune cell phenotypes within the breast tumor microenvironment, offering a better understanding of cancer progression and immune therapy responses (239). In addition, breakthroughs in AI-driven therapeutic target exploration support drug discovery and development, further advancing the field (240, 241).

Nevertheless, several key challenges remain for AI applications in organoid research. First, sample sizes are often limited, particularly in high-throughput single-cell or multi-omics experiments. Insufficient data may lead to inadequate coverage for model training, making it difficult to capture features of rare cell types or complex cellular states and thereby reducing predictive accuracy and generalizability (242–244). Second, batch effects across culture batches, imaging conditions, or experimental platforms can introduce systematic biases that obscure true biological signals and compromise the accurate identification of cellular phenotypes, molecular features, or dynamic responses. Accordingly, algorithmic correction and/or standardized workflows are required to mitigate these confounders (245, 246). Finally, obtaining reliable ground-truth labels in three-dimensional organoid systems remains challenging. Owing to tissue thickness and optical constraints, fluorescent labeling or sectioning may not fully capture internal spatial architecture, leading to missing information in training datasets and increasing the complexity of model validation and interpretation. In addition, limitations in 3D live imaging and labeling technologies restrict the ability of AI models to precisely simulate organoid development and intercellular interactions (247).

Despite these challenges, AI shows great potential in image analysis and dynamic monitoring of organoids. It can precisely diagnose diseases by analyzing large volumes of images, providing technical support for organoid image analysis (248–250). Deep learning (DL) technologies further enhance image analysis. Timothy Kassis’ team created and publicly released a unique dataset that allows AI to fully automate the analysis of thousands of images for human intestinal organoid localization and quantification (251). The following year, Rutger N U Kok’s team developed a semi-automated cell tracker, making it possible to track cells within organoids and directly observe patterns of cell division and differentiation (252). Various machine learning algorithms and deep learning architectures could be used for compound screening and toxicity testing. This would enhance the effectiveness and precision of research, as well as introducing new approaches to drug discovery (26). In organoid immuno-microenvironment studies, AI can assist with single-cell gene expression and immune repertoire analysis (25). Furthermore, DL has a positive impact on improving gynecological cancer screening and diagnosis under resource-limited conditions (253). Additionally, the combination of MRI with advanced automated image analysis tools supports long-term monitoring, analysis, and clinical applications of brain organoids (254). Based on these advancements, we can reasonably infer that AI holds potential in assisting gynecological inflammation-related organoid research, offering new paths for research and treatment in this field (158). In the future, efforts should be made to promote the deep integration of organoids and AI, with animal models serving as auxiliaries, to construct standardized, vascularized, and immune co-cultured multi-organ interaction models, addressing their systemic limitations. At the same time, a unified quality control and regulatory system should be established to promote the complementary advantages of organoids, AI, and animal models, providing an efficient, ethical, and translatable new generation of research paradigms for precision medicine and new drug development.

### 4.3 AI-assisted study of inflammasomes in organoids

Inflammasomes are critical multi-protein complexes involved in innate immunity. They recognize intracellular pathogen-associated molecular patterns (PAMPs) and damage-associated molecular patterns (DAMPs), promoting the maturation and release of inflammatory factors such as IL-1β and IL-18 by activating caspase-1. Inflammasomes also induce pyroptosis, which plays a key role in the body’s defense mechanisms (6, 7). In recent years, studies have demonstrated that inflammasomes play a significant role in a variety of pathological conditions, including infectious diseases, metabolic disorders, autoimmune diseases, tumors, and chronic inflammation (255–257). In gynecology, inflammasomes contribute to multiple inflammatory conditions. NLRP3 and IFI16 show connections to pelvic inflammatory disease, endometriosis, cervicitis, and HPV-associated cervical cancer (84, 93, 258–260).

But traditional two-dimensional cell models can’t really copy the complexity of the inflammatory microenvironment seen in gynaecological diseases. These models don’t have a true three-dimensional structure and can’t show the relationship between cells and the stuff around them accurately. They don’t show how all the different cells in the inflamed area are interacting with each other (261–263). Organoid models better mimic the female reproductive system. They are well-suited for inflammasome research in gynecological diseases (169, 263). For example, in a co-culture model of endometriotic cells with other cell types, organoids have been used to study the early stages of endometriosis, including initial adhesion, invasion, and angiogenesis (43). A mouse organoid study showed how AIM2 and NLRP3 inflammasomes drive inflammation through IL-1β. This process actively promoted angiogenesis, cell adhesion, and tissue growth. blocking IL-1β effectively suppressed these effects, leading to improved vascular outcomes (264). A novel, laboratory-developed, mouse-human endometrial organoid model demonstrated the physiological responses of the endometrial epithelium to estrogen and progesterone, including increased cell proliferation in response to estrogen and maturation in response to progesterone, which lasted for more than 14 days (265). These models help to investigate how oestrogen influences NLRP3 inflammasome levels. They offer a more accurate representation of the human inflammatory environment, allowing researchers to study cell interactions influenced by inflammasomes in co-culture models (266). Thus, AI plays a vital role in the analysis of inflammasomes in these organoid models, enabling deeper insights into their regulation mechanisms and their association with the progression of gynecological diseases (218, 267–269).

#### 4.3.1 Integration with transcriptomics

Transcriptomics is the study of all RNA molecules in cells, tissues, or organisms (270, 271). RNAsequencing (RNA-seq) has become the field’s principal workhorse, enabling rapid, comprehensive measurement of transcript identity and abundance in a given specimen (272). When paired with AI-guided analytics, researchers can better understand the mechanistic details of inflammasome activation through transcriptomic analysis (28, 106, 273, 274).

AI algorithms excel at extracting biologically meaningful features from high-dimensional data generated by RNA-seq (107, 273, 275–277). ML can identify differentially expressed genes (DEGs), such as PYCARD (ASC), IL1B, and IL18, which are linked to key inflammasome pathways in gynecological inflammation, such as NLRP3 and AIM2. On this basis, by further constructing predictive models for gynecological organoids, it is possible to determine whether cells or tissues are in a state of inflammasome activation (278–280). DL, coupled with RNA-seq, supports representation learning and pattern discovery in organoid transcriptomes, providing dual support for pathway mechanism analysis (281, 282). NLP can further quantify cell states and gene functions by constructing embedding vectors, making it a valuable tool for studying inflammasome activation in organoids (107). Combining AI with RNA-seq enables accurate and efficient computational frameworks for identifying the mechanisms of inflammasome activation in gynecological diseases.

#### 4.3.2 Binding to the proteome

The integration of AI technology can break through the limitations of traditional proteomics analysis, providing a powerful tool for the precise analysis of inflammasomes in organoids. Traditional proteomics techniques, such as liquid chromatography-tandem mass spectrometry (LC-MS/MS) and two-dimensional polyacrylamide gel electrophoresis (2D-PAGE), enable protein identification and quantification (283–285).

However, such techniques have problems such as complex data processing, difficult to detect low-abundance proteins, and low efficiency of post-translational modification capture. Moreover, the experimental process is time-consuming and the interpretation of results is highly dependent on manual work, which limits their application efficiency in fine mechanism research (286–288).

AI-assisted proteomics significantly optimizes data processing and analysis, improving the research efficiency and accuracy of results. For instance, DISCO-MS, which combines tissue transparency, deep learning-based image analysis, automated tissue extraction, and mass spectrometry (289), offers an unbiased path for analyzing inflammasomes such as NLRP1, NLRP3, NLRC4, and AIM2. Additionally, AI tools like π-HuB (Human proteomics Navigator) use high-resolution proteomics to analyze the human proteome, offering powerful insights into inflammasome research in organoid models (290).

#### 4.3.3 Integration with the metabolome

Inflammasomes play a critical role in gynecological inflammation, which can be triggered by microbial infections, leading to inflammatory responses that regulate immune responses. The interaction between inflammasomes and microbial metabolites influences the development of gynecological diseases (291).

**Figure 3:**
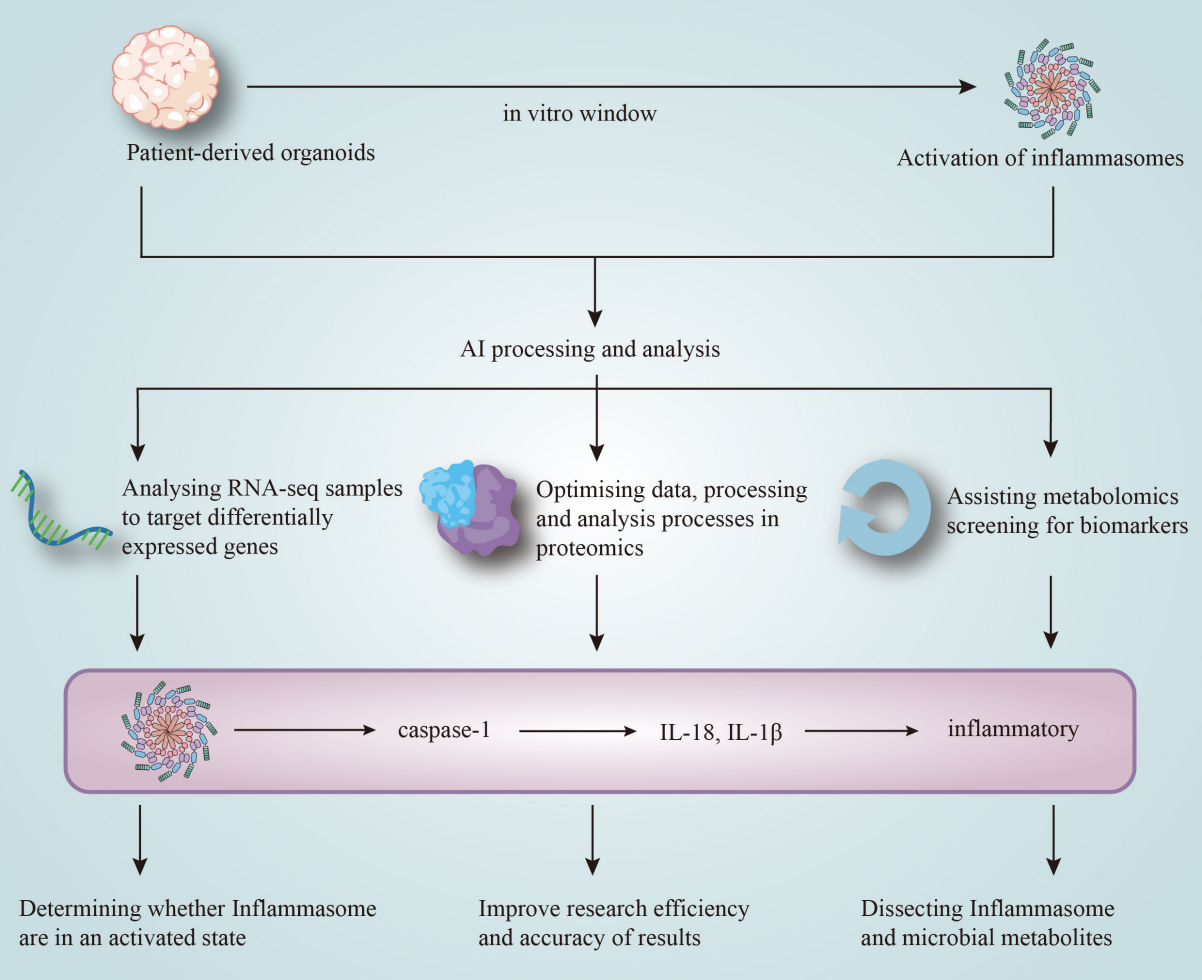
AI-integrated analysis of inflammasome-related mechanisms in gynecological inflammation through transcriptomics, proteomics, and metabolomics.

With the continuous advancement of medical research, new technologies and methods provide novel approaches for precise diagnosis and treatment of diseases. In the study of bacterial vaginosis (BV), machine learning-based diagnostic models can address limitations such as subjectivity in diagnosis, technical constraints, and the inability to comprehensively reflect the vaginal microecological status. In recent years, the combination of multi-omics and machine learning (ML) has also provided strong support for the screening and validation of BV-related biomarkers (111). The advancement of high-throughput sequencing technologies has opened new avenues for exploring the microbiome and inflammasomes (292–294). Among them, the unsupervised learning framework NMFGOT can effectively integrate microbiome and metabolome data, handle complex nonlinear relationships, and facilitate downstream biological analysis (27). In other areas of medical research, such as analyzing the urinary metabolomic changes in pregnant women to predict gestational age (GA) and delivery time, or exploring the interactions between gut microbiota and metabolites, various statistical and ML methods are commonly employed. These are often combined with 16S rRNA gene sequencing and LC-MS/MS metabolomics, complemented by network module analysis to uncover potential associations and construct clinical prediction models (295, 296). The DeepMSProfiler method based on deep learning (DL) can effectively address challenges such as complex data processing, batch-to-batch variation, and unknown metabolites, thus improving the accuracy and reliability of the output results (297). To gain a deeper understanding of the microbial ecosystem in complex gynecological organoids, ecology-based computational methods such as GutCP can predict untested cross-feeding interactions within the microbiome (298). These AI-based metabolomics analysis strategies provide valuable approaches for elucidating the interaction mechanisms between inflammasomes and microbial metabolites in gynecological inflammation (**Figure 3**).

## 5 Conclusion and discussion

The inflammasome serves as a pivotal regulatory hub for immune activation and inflammatory cell death within the female reproductive system, linking diverse stimuli such as infection, metabolic stress, and hormonal imbalance to disease progression. In conditions including endometriosis, ovarian cancer, polycystic ovary syndrome (PCOS), and premature ovarian insufficiency, aberrant inflammasome activation drives persistent inflammation, fibrosis, and immune evasion, thereby propelling pathological processes. These findings establish inflammasomes as pivotal molecular players in chronic reproductive inflammation. However, translating these mechanistic discoveries into effective clinical interventions remains profoundly challenging, fundamentally due to the inability of traditional two-dimensional culture systems and animal models to authentically replicate the intricate immunological and endocrine interactions within human reproductive tissues.

To overcome this obstacle, we have proposed a new approach that integrates the analysis of inflammasome mechanisms, the construction of organoid models, and artificial intelligence (AI) technology. Firstly, organoids derived from patients’ endometrium, ovaries, and cervix can reconstruct the three-dimensional structure and multi-cellular complexity of tissues, simulate hormone responses and inflammatory signaling pathways, providing a physiologically relevant platform for mechanism research and drug screening. At the same time, this process generates massive, high-dimensional, and multi-modal data. Subsequently, the introduction of computational tools such as AI can efficiently process and analyze the complex datasets generated by organoid research. This is because AI can utilize deep learning, computer vision, and multi-omics integration to quantify the dynamic changes in inflammasome activation, identify potential biomarkers, and predict treatment responses in different disease states.

This three-in-one integrated approach has been demonstrated in various studies. Dana El Soufi El Sabbagh et al. aimed to further clarify whether bipolar disorder (BD) is accompanied by mitochondrial dysfunction and enhanced inflammatory reactivity. They used iPSC-derived brain organoids (CO) from BD patients to simulate more physiologically relevant disease-specific metabolic and inflammatory dysfunctions and proved the increased sensitivity of NLRP3 inflammasome activation through metabolomics and other methods. Shashi Kant Tiwari et al. constructed iPSC-derived lung organoids (LORGs) as an *in vitro* model system to study the pathogenesis of the novel coronavirus SARS-CoV-2. By examining host response genes, they found that key genes in the inflammasome pathway, such as NLRP3, were upregulated, indicating that SARS-CoV-2 infection of LORGs activated genes involved in the inflammasome pathway (NLRP3, etc.). Thus, this three-in-one integrated approach is feasible, and its application in the precise diagnosis and treatment of gynecological diseases also holds promise.

It is worth noting that although the interdisciplinary research progress of this method is rapid, it is still in its early stages and faces several key limitations.Current inflammasome research in gynaecological diseases predominantly focuses on fundamental mechanisms, with differences between disease subtypes and their dynamic associations with the hormonal cycle is still lacking. While organoid models effectively reproduce tissue architecture and certain immune features, they lack complete immune cell populations and the humoral environment, hindering precise simulation of the complex regulation of chronic inflammation and immune tolerance. AI−driven analyses, despite clear promise for multi−omics integration and disease prediction, are frequently hampered by small cohort sizes, marked inter−dataset heterogeneity, and restricted interpretability. These limitations impact the clinical reliability and generalisability of models.

Looking ahead, future research should further advance interdisciplinary integration by establishing high-fidelity gynaecological organoid systems incorporating immune, endocrine, and metabolic components. This should be combined with integrated analyses using large-scale patient-derived samples and longitudinal clinical data. Concurrently, developing interpretable AI algorithms is essential to achieve dynamic visualisation and causal inference within inflammasome regulatory networks. From a clinical perspective, urgent, multicentre validation is essential to confirm the safe and effective use of inflammasome-targeting therapeutics and AI predictive models in the management of gynaecological diseases. Collectively, synergistic research integrating inflammasomes, organoids, and artificial intelligence is charting novel pathways in female reproductive immunology. As model precision and algorithmic transparency advance, this field holds promise for genuine translation from mechanism discovery to clinical application, laying a robust foundation for precision diagnosis and personalised treatment of gynaecological disorders.
